# Antigen Recognition By Autoreactive Cd4^+^ Thymocytes Drives Homeostasis Of The Thymic Medulla

**DOI:** 10.1371/journal.pone.0052591

**Published:** 2012-12-27

**Authors:** Magali Irla, Lucia Guerri, Jeanne Guenot, Arnauld Sergé, Olivier Lantz, Adrian Liston, Beat A. Imhof, Ed Palmer, Walter Reith

**Affiliations:** 1 Department of Pathology and Immunology, University of Geneva Medical School, Geneva, Switzerland; 2 Département de Biologie des Tumeurs and Inserm U932, Institut Curie, Paris, France; 3 Autoimmune Genetics Laboratory, VIB and University of Leuven, Leuven, Belgium; 4 Experimental Transplantation Immunology, Department of Biomedicine, University Hospital-Basel, Basel, Switzerland; Oklahoma Medical Research Foundation, United States of America

## Abstract

The thymic medulla is dedicated for purging the T-cell receptor (TCR) repertoire of self-reactive specificities. Medullary thymic epithelial cells (mTECs) play a pivotal role in this process because they express numerous peripheral tissue-restricted self-antigens. Although it is well known that medulla formation depends on the development of single-positive (SP) thymocytes, the mechanisms underlying this requirement are incompletely understood. We demonstrate here that conventional SP CD4^+^ thymocytes bearing autoreactive TCRs drive a homeostatic process that fine-tunes medullary plasticity in adult mice by governing the expansion and patterning of the medulla. This process exhibits strict dependence on TCR-reactivity with self-antigens expressed by mTECs, as well as engagement of the CD28-CD80/CD86 costimulatory axis. These interactions induce the expression of lymphotoxin α in autoreactive CD4^+^ thymocytes and RANK in mTECs. Lymphotoxin in turn drives mTEC development in synergy with RANKL and CD40L. Our results show that Ag-dependent interactions between autoreactive CD4^+^ thymocytes and mTECs fine-tune homeostasis of the medulla by completing the signaling axes implicated in mTEC expansion and medullary organization.

## Introduction

The thymus ensures the generation of a self-tolerant T cell receptor (TCR) repertoire. Tolerance to self-antigens (Ags) is established in the medulla, a specialized microenvironment mainly composed of medullary thymic epithelial cells (mTECs) and dendritic cells (DCs). mTECs are critical for inducing self-tolerance because they constitute a thymic reservoir of numerous peripheral tissue-restricted self-Ags (TRAs) 1,2. Defective TRA expression results in autoimmunity 3. mTECs can present TRAs to autoreactive CD8^+^ and CD4^+^ SP thymocytes to promote their deletion or the generation of natural regulatory T cells 4,5,6. TRAs expressed by mTECs are also captured by thymic DCs, which help to purge the thymocyte repertoire of autoreactive TCR specificities 4. Negative selection is further reinforced by circulating DCs displaying self-Ags captured in the periphery 7. The medulla thus ensures the establishment of T-cell tolerance via tight collaboration between mTECs and DCs 8.

Mice presenting a block in thymocyte development at the double-positive (DP) stage exhibit small scattered medullary islets 9,10,11, indicating that medulla formation requires SP thymocytes 12. SP thymocytes and mTECs thus engage in a bidirectional “crosstalk” that controls formation and organization of the medulla 12,13 as well as thymocyte deletion. We recently discovered that autoreactive CD4^+^ thymocytes play a privileged role in governing the development of the mTEC subset that displays a fully mature phenotype, which constitutes approximately 20% of the total mTEC population 14. However, the nature of the cellular interactions and molecular mechanisms that controls development and architectural organization of the entire medullary compartment remains poorly understood.

Three members of the tumor necrosis factor receptor superfamily and their ligands have been implicated in mTEC development 13,14,15,16,17,18. Cooperation between the engagement of receptor activator of nuclear factor Kappa B (RANK) and CD40 on mTECs by RANKL and CD40L expressed by SP thymocytes determines mature mTEC cellularity in adult mice 16 while lymphotoxin β receptor (LTβR) and LTα1β2 (LT) influence medulla organization 19,20,21. However, the respective roles of these three signaling axes in medulla development, and the sequence in which they operate, remain elusive.

We demonstrate here that conventional autoreactive CD4^+^ thymocytes are essential and sufficient for fostering total mTEC expansion and determining the 3-dimensional (3D) organization of the entire medullary compartment. This critical function of autoreactive CD4^+^ thymocytes is mediated by Ag-dependent interactions with mTECs displaying cognate Ag-MHCII complexes and engagement of the CD28-CD80/86 costimulatory axis. This crosstalk induces LTα expression in the CD4^+^ thymocytes and RANK in mTECs, thereby completing two of the three signaling axes regulating medulla formation and organization. Finally, we show that the crosstalk between autoreactive CD4^+^ thymocytes and mTECs is a dynamic homeostatic process that governs the remarkable plasticity of the postnatal medulla, allowing it to adapt its size and organization to the output of autoreactive thymocytes.

## Materials And Methods

### Mice

Mice were on a C57BL/6 background, specific-pathogen-free and sacrificed at 5–8 weeks of age. *β2m* ^−/−^ 22, *H2-Aα* ^−/−^ 23, *CIIta* ^IV-/IV-^ 24, *TCRα* ^−/−^ 25, *Rag2* ^−/−^ 26, *Marilyn*:*Rag2* ^−/−^ 27, *3BBM74:Rag2* ^−/−^ 28, *B3K508:Rag1* ^−/−^ 29, LTα^−/−^ 30, CD80/86^−/−^ 31, CD28^−/−^ 32 and Foxp3DTR 33 mice have been described. RIP-mOVA 34 and OTII 35 mice were backcrossed onto a *Rag2* ^−/−^ background. All animal procedures were performed in accordance with the Institutional Ethical Committee of Animal Care in Geneva and Cantonal Veterinary Office. The Institutional Ethical Committee of Animal Care in Geneva and Cantonal Veterinary Office specifically approved this study through the experimentation ID: 1005-3697-3.

### Bone Marrow Chimeras

Irradiated (900 rad) WT male recipients were injected i.v. with 5×10^6^ bone marrow cells from WT and/or Marilyn:Rag2^−/−^ males.

### Thymic Sections

Frozen sections cut at 20-µm of thickness were stained with rabbit-anti keratin 14 (Covance) and rat-anti keratin 8 (abcam) and detected using Cy3-conjugated anti-rabbit IgG (Invitrogen) and Alexa Fluor 488-conjugated anti-rat IgG (Invitrogen), respectively. Sections were counterstained with DAPI and mounted with Mowiol (Calbiochem). Medullary areas were measured using Metamorph. For 3D reconstructions, medullas were delimited based on K14 staining using Matlab. Medullary volumes were computed using Imaris. Hematoxylin-Eosin (HE) stained sections (5-µm) were prepared from fixed (4% formaldehyde) paraffin-embedded thymuses. Images were acquired with a LSM 510 confocal microscope or a Mirax Midi scanner (Zeiss).

### Tec Isolation And Flow Cytometry

TECs were prepared as described 14. Hematopoietic cells were depleted with anti-CD45 magnetic beads (Miltenyi Biotec) by AutoMACS. TECs and thymocytes were analyzed by FACS using a FACScalibur or a LSRII (Becton Dickinson). Cells were incubated for 15 min at 4°C with Fc-block (anti-CD16/CD32, PharMingen) before staining with the following antibodies: anti-CD45 (30-F11), anti-EpCAM (G8.8), anti-Ly51 (6C3), anti-CD80 (16-10A1), anti-I-Ab (AF6-120.1) and anti-LTα (AF.B3); all purchased from Pharmingen except for anti-EpCAM that was purchased from eBioscience. For intracellular Aire (5H12, eBioscience) and Ki67 (B56, PharMingen) stainings, cells were fixed and stained with BD Cytofix/Cytoperm and Perm/Wash (Biosciences). Gating on mTECs was performed as described 14.

### Thymic Organ Culture

Thymic lobes from E15.5 C57BL/6 embryos were treated with 1.35 mM 2′-deoxiguanosine (2-dGUO, Sigma) for 6 days. For FTOC, 2-dGUO-treated lobes were cultured for 4 days in DMEM supplemented with 2 µg/ml RANKL (R&D Systems), 5 µg/ml CD40L (R&D Systems) and/or 2 µg/ml agonistic anti-LTβR antibody (clone 4H8) 36. RTOCs were performed as described 37. 2-dGUO treated lobes were disaggregated to obtain thymic stroma. CD4^+^CD8^+^βTCR^lo/int^HSA^hi^ thymocytes were sorted from 3 week old *OTII:Rag2* ^−/−^ mice. Stromal cells and thymocytes were mixed in a 1∶3 ratio and cultured as standing drops. After 5 days, RTOCs were digested to single cell suspensions for flow cytometry analysis.

### Rt-Pcr

Real-time RT-PCR was performed as described 14. GAPDH mRNA was used for normalization. Primer sequences are available upon request.

### Thymocyte Purification And Activation

Thymocytes were incubated at 4°C with Fc-block, then with anti-CD4 (RM4-5, Pharmingen) and anti-CD8a antibodies (53-6.7, Pharmingen). DN, DP and SP thymocytes were sorted with a FACSVantage (Becton Dickinson). Cell purity was more than 90%. Thymocytes were stimulated for 24 h in RPMI with 1 µg/ml anti-CD28 antibodies (37.51, Pharmingen) in 96 well plates pre-coated with 2 µg/ml of anti-CD3 antibodies (145-2C11, Pharmingen), or by co-culture with OVAp-loaded mTECs.

### Peptide Injection

4–9 week old mice were injected i.v. with 85 nmol of OVAp (OVA_323–339_, ISQAVHAAHAEINEAGR) or H-Yp (NAGFNSNRANSSRSS).

### Statistics

Statistical significance was assessed by two-tailed Student's t test: ***, p<0.001; **, p<0.01; *, p<0.05.

## Results

### Cd4^+^ Thymocytes Are Critical For Sustaining Medulla Formation

**Figure 1:**
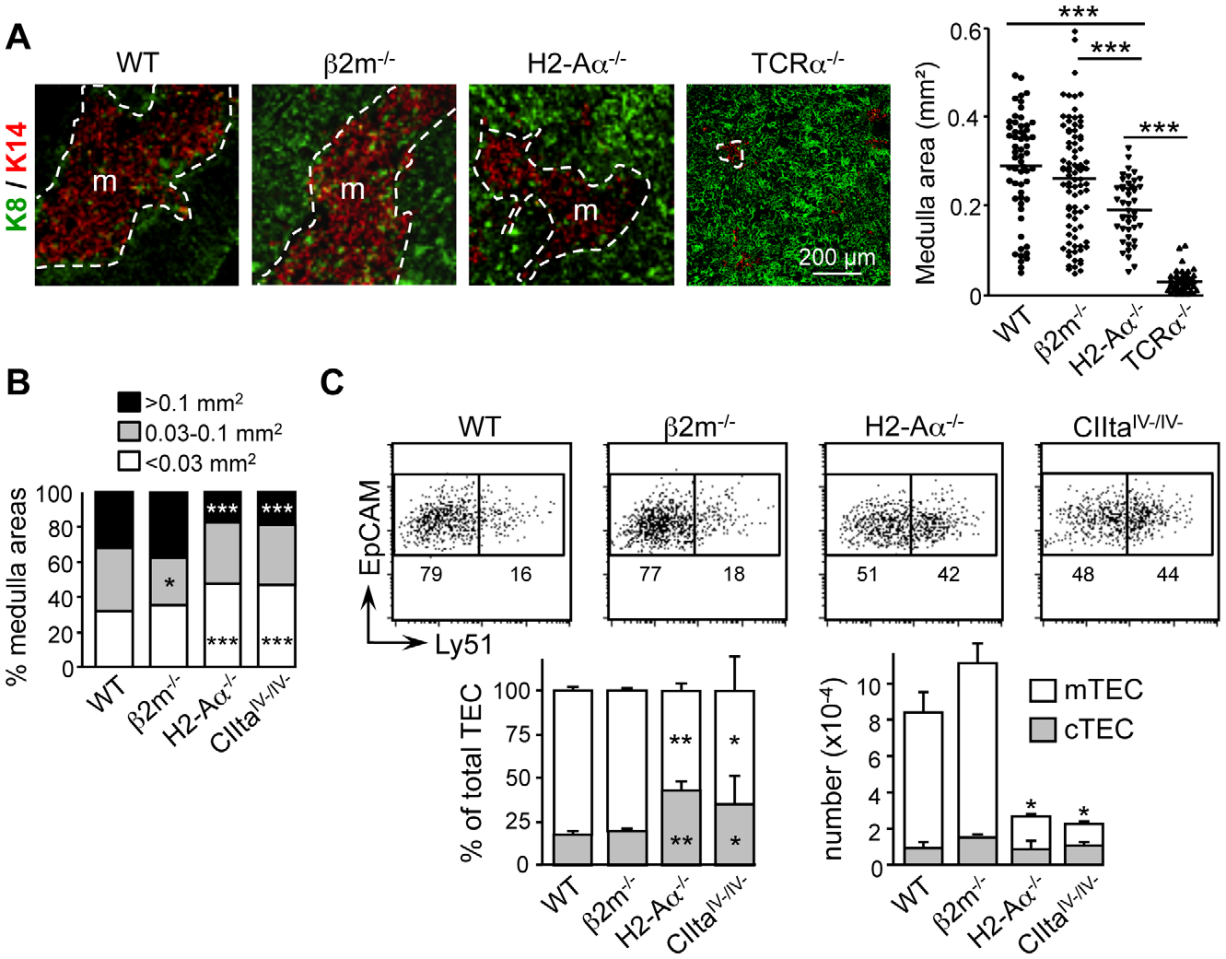
Medulla formation is defective in mice lacking CD4^+^ thymocytes. (A) Sections from WT, *β2m* ^−/−^, *H2-Aα* ^−/−^ and *TCRα* ^−/−^ thymi were stained with antibodies against K14 and K8; m, medulla. The graph shows medullary areas obtained from 3 experiments: symbols represent individual confocal images; lines represent medians. (B) The distribution of medullary areas (mm^2^) counterstained with HE is shown for WT, *β2m* ^−/−^, *H2-Aα* ^−/−^ and *CIIta* ^IV-/IV-^ mice: 3 mice per genotype; number of sections is 87 for WT, 90 for *β2m* ^−/−^, 91 for *H2-Aα* ^−/−^ and 78 for *CIIta* ^IV-/IV-^; significance relative to WT. (C) Representative FACS profiles are shown (top) for Ly51 expression by CD45^−^EpCAM^+^ TECs from WT, *β2m* ^−/−^, *H2-A*α^−/−^ and CIIta^IV-/IV-^ mice: percentages of cells are indicated. Percentages (bottom left) and numbers per thymus (bottom right) of CD45^−^EpCAM^+^Ly51^−/lo^ mTECs and CD45^−^EpCAM^+^Ly51^+^ cTECs are shown: means and SD from 3 measurements; significance relative to WT.

We had previously reported that CD4^+^ thymocytes development is critical for determining the cellularity of mature Aire^+^ mTECs 14. However, it was not known whether CD4^+^ thymocytes also play a privileged role in governing total mTEC cellularity and/or patterning of the medulla. To address this question, thymic sections from wild-type (WT), *β2m* ^−/−^ (lacking CD8^+^ thymocytes), *H2-Aα* ^−/−^ (lacking CD4^+^ thymocytes) and *TCRα* ^−/−^ (lacking SP thymocytes) mice were stained with antibodies directed against the cortical TEC (cTEC) marker keratin 8 (K8) and the mTEC marker keratin 14 (K14) (Figure 1A). Medullary areas were larger in both *β2m* ^−/−^ and *H2-Aα* ^−/−^ mice compared to *TCRα* ^−/−^ mice, which contain only rudimentary medullas 11,17,38. However, whereas medullary areas were of WT size in *β2m* ^−/−^ mice, they were markedly underdeveloped in *H2-Aα* ^−/−^ mice. We also quantified medullary areas by HE staining of thymic sections from WT, *β2m* ^−/−^, *H2-Aα* ^−/−^ and *CIIta* ^IV-/IV-^ mice (Figure 1B). Like *H2-Aα* ^−/−^ thymi, *CIIta* ^IV-/IV-^ thymi lack CD4^+^ thymocytes 39. Medullary size distributions were strongly skewed towards smaller sizes in *H2-Aα* ^−/−^ and *CIIta* ^IV-/IV-^ mice but similar to WT in *β2m* ^−/−^ mice. Consistently, the medullary volume was also reduced in *H2-Aα* ^−/−^ mice compared to WT and *β2m* ^−/−^ mice (data not shown).

Significant reductions in total mTEC frequencies and matching increases in cTEC frequencies were evident in *H2-Aα* ^−/−^ and *CIIta* ^IV-/IV-^ mice (Figure 1C). In contrast, mTEC and cTEC frequencies were similar to WT in *β2m* ^−/−^ mice. Absolute mTEC numbers were strongly reduced in *H2-Aα* ^−/−^ and *CIIta* ^IV-/IV-^ thymi, whereas cTEC numbers were unchanged (Figure 1C). Reductions in mTEC numbers in *H2-Aα* ^−/−^ and *CIIta* ^IV-/IV-^ mice affected both mature (Aire^+^, CD80^hi^) and immature (CD80^int^) mTEC subsets (Figure S1A).

Defective medulla formation in the absence of CD4^+^ thymocytes was not associated with altered DC populations. Frequencies and numbers of plasmacytoid DCs (CD11c^int^PDCA1^+^), migrating cDCs (CD11c^hi^CD8*α* ^lo^Sirpα^hi^) and intrathymic cDCs (CD11c^hi^CD8*α* ^hi^Sirpα^lo^) were similar in WT, *β2m* ^−/−^, *H2-Aa* ^−/−^ and *CIIta* ^IV-/IV-^ mice (Figure S1B). No difference in medullary localization of DCs was observed (Figure S1C).

These results demonstrate that CD4^+^ thymocytes are required and sufficient for sustaining medulla formation by controlling total mTEC cellularity. This selective dependence on CD4^+^ thymocytes is specific of mTECs, as a block in CD4^+^ thymocyte development has no adverse effects on DCs.

### Ag-Specific Interactions With Cd4^+^ Thymocytes Control Medulla Formation And Organization

**Figure 2:**
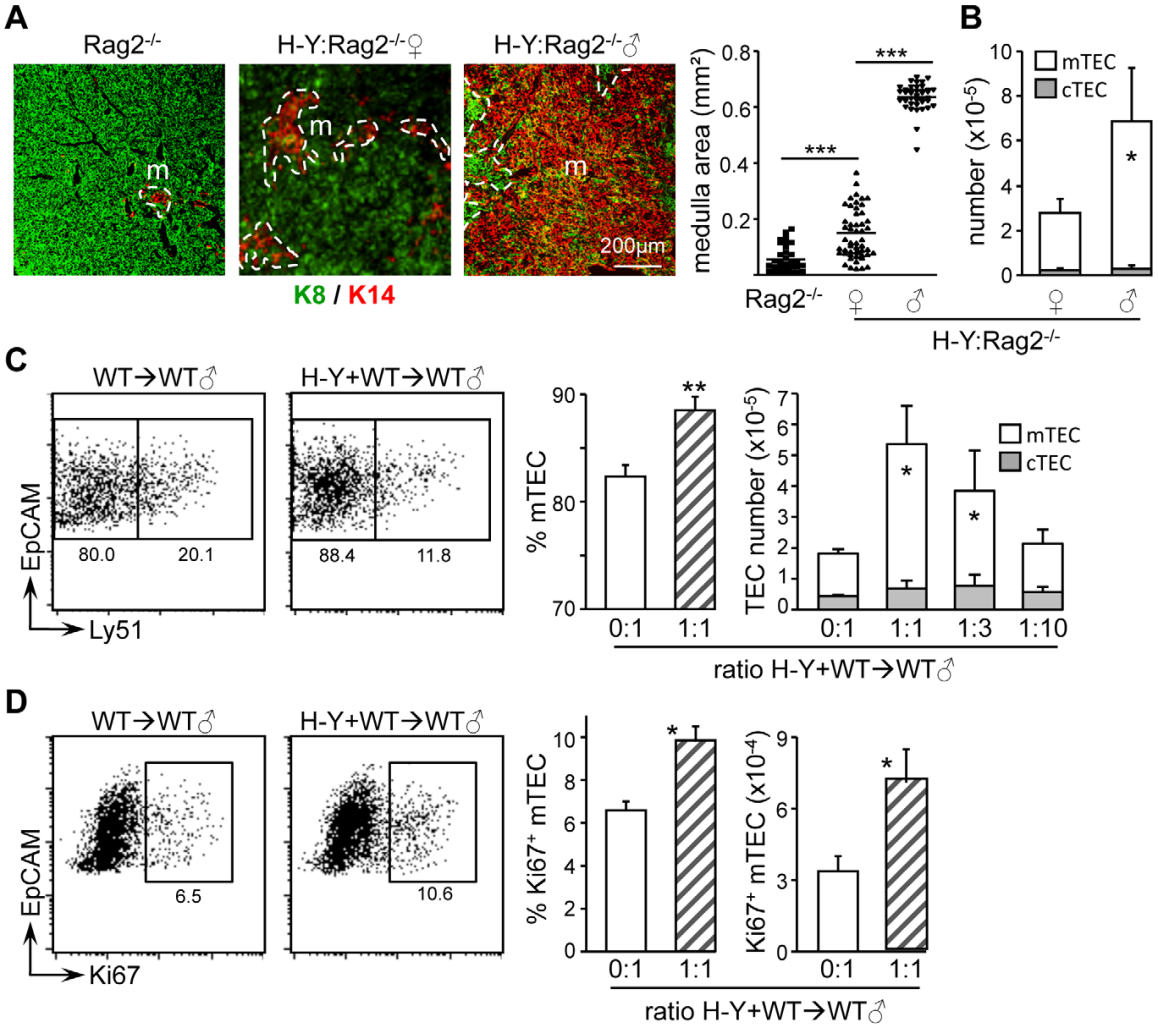
Medulla formation is controlled by autoreactive CD4^+^ thymocytes. (A) Thymic sections from *Rag2* ^−/−^ mice and *Marilyn:Rag2* ^−/−^ females or males were stained with antibodies against K8 and K14: m, medulla. The graph shows quantifications of medullary areas: symbols represent individual confocal images; lines represent medians; data from 3 experiments, each with 2–3 mice per group. (B) The graph shows numbers per thymus of CD45^−^EpCAM^+^Ly51^−/lo^ mTECs and CD45^−^EpCAM^+^Ly51^+^ cTECs in *Marilyn:Rag2* ^−/−^ females and males: means and SD from 3 experiments. (C) Representative FACS profiles are shown for Ly51 expression by CD45^−^EpCAM^+^ TECs from WT→WT and mixed H-Y+WT→WT (1∶1 ratio) chimeras. Graphs show percentages of CD45^−^EpCAM^+^Ly51^−/lo^ mTECs and numbers per thymus of CD45^−^EpCAM^+^Ly51^−/lo^ mTECs and CD45^−^EpCAM^+^Ly51^+^ cTECs for chimeras prepared with the indicated H-Y∶WT BM ratio: means and SD derived from 3 measurements; significance relative to WT→WT chimeras (0∶1 ratio). (D) Representative FACS profiles are shown for Ki67 expression by CD45^−^EpCAM^+^Ly51^−/lo^ mTECs from WT→WT and mixed H-Y+WT→WT (1∶1 ratio) chimeras. Graphs show percentages and numbers per thymus of Ki67^+^ mTECs for chimeras prepared with the indicated H-Y∶WT BM ratio: means and SD from 3 measurements; significance relative to WT→WT chimeras (0∶1 ratio).

We previously reported that the development mature Aire^+^ mTECs is fostered by Ag-specific interactions with CD4^+^ thymocytes 14. TCR transgenic mice were therefore used to determine whether CD4^+^ thymocyte driven medulla formation and organization also require Ag-specificity. We first compared medullas between male and female *Marilyn*:*Rag2* ^−/−^ mice, which carry an MHCII-restricted TCR recognizing the male-specific H-Y Ag 27. In these mice, CD4^+^ thymocytes are deleted in males but not in females 14. Thymic sections from *Rag2* ^−/−^ mice and *Marilyn*:*Rag2* ^−/−^ females or males were stained for K8 and K14 (Figure 2A). Medullary areas were enlarged only modestly in *Marilyn*:*Rag2* ^−/−^ females compared to *Rag2* ^−/−^ mice, but increased dramatically in *Marilyn*:*Rag2* ^−/−^ males. This strong medullary expansion was accompanied by a marked increase in mTEC numbers (Figure 2B).

The role of autoreactive CD4^+^ thymocytes in driving mTEC cellularity was further assessed by generating mixed BM chimeras in which irradiated WT males were reconstituted with variable mixtures of BM from WT and *Marilyn*:*Rag2* ^−/−^ males (Figure 2C). Reconstitution with *Marilyn*:*Rag2* ^−/−^ BM increased mTEC cellularity to greater than WT levels in a dose dependent manner. This was associated with a significant increase in proliferating Ki67^+^ mTECs (Figure 2D), suggesting that autoreactive CD4^+^ thymocytes amplify mTEC cellularity directly by stimulating their proliferation.

**Figure 3:**
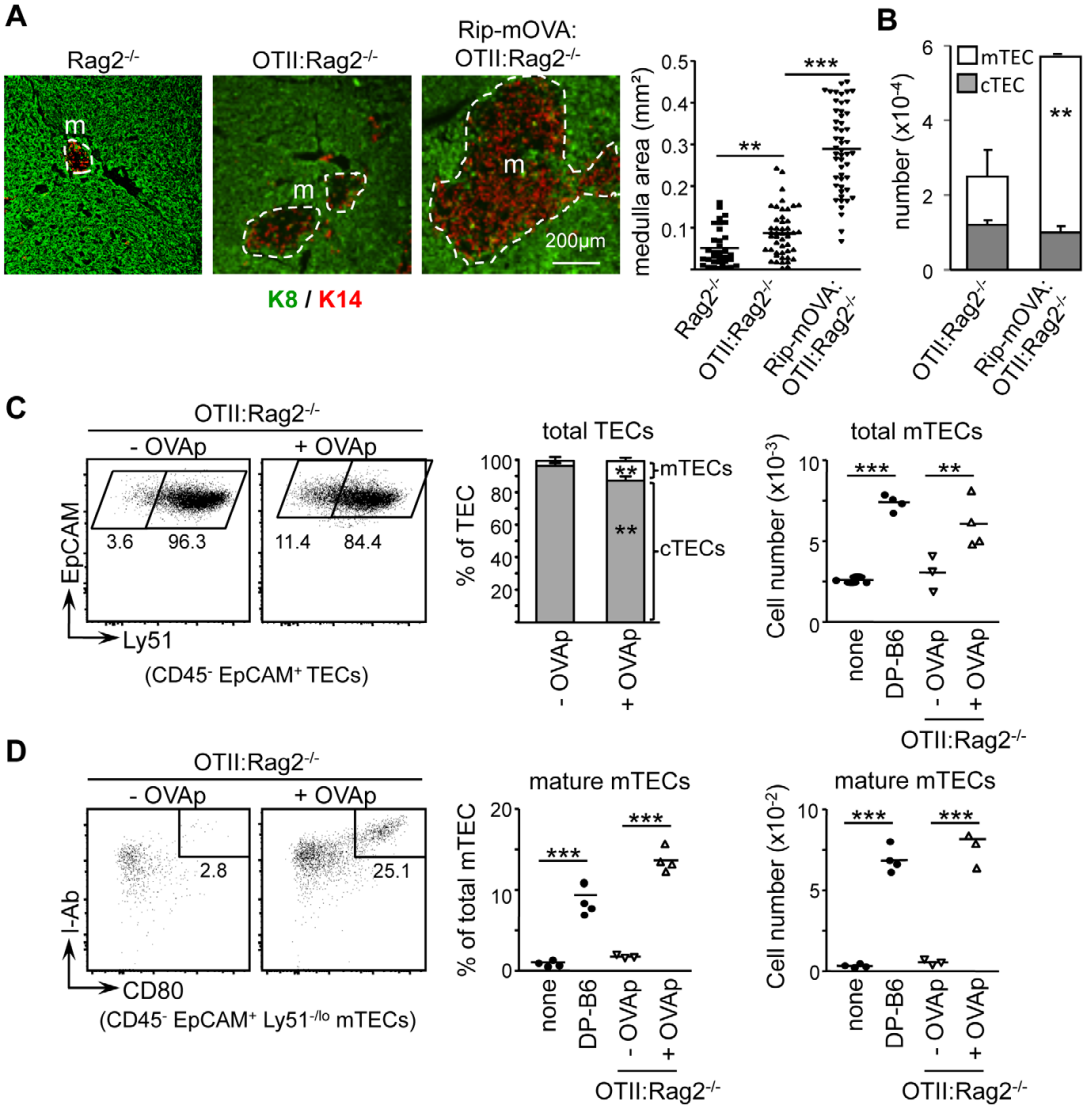
mTEC cellularity is controlled by Ag-specific interactions with CD4^+^ thymocytes. (A) Sections from *Rag2* ^−/−^, *OTII:Rag2* ^−/−^ and *Rip-mOVA:OTII:Rag2* ^−/−^ thymi were stained with antibodies against K8 and K14: m, medulla. The graph shows quantifications of medullary areas: symbols represent individual confocal images; lines represent medians; data from 3 experiments, each with 2–3 mice per group. (B) The graph shows numbers per thymus of CD45^−^EpCAM^+^Ly51^−/lo^ mTECs and CD45^−^EpCAM^+^Ly51^+^ cTECs: means and SD from 3 measurements; significance relative to WT. (C) RTOCs using *OTII:Rag2* ^−/−^ DP thymocytes were cultured for 5 days with (+) or without (−) OVAp. Control cultures contained no thymocytes (none) or WT DP thymocytes (DP-B6). Representative FACS profiles are shown for Ly51 expression by CD45^−^EpCAM^+^ TECs: percentages of cells are indicated. Graphs show frequencies of EpCAM^+^Ly51^+^ cTECs and EpCAM^+^Ly51^−/lo^ mTECs (left) or numbers of EpCAM^+^Ly51^−/lo^ mTECs (right). (D) Representative FACS profiles are shown for the expression of I-Ab and CD80 by mTECs in RTOCs cultured with (+) or without (−) OVAp: percentage of cells are indicated. Graphs show frequencies and numbers of mature I-Ab^hi^CD80^hi^ mTECs: data from 4 experiments, each with 3–5 RTOCs per condition.

We next studied medulla formation in *OTII:Rag2* ^−/−^ mice, which express an MHCII-restricted TCR specific for ovalbumin (OVA), and *OTII:Rag2* ^−/−^ mice carrying a *RIP-mOVA* transgene driving the synthesis of membrane-bound OVA in mTECs 40. Medullary areas were increased only slightly in *OTII:Rag2* ^−/−^ mice relative to *Rag2* ^−/−^ controls. They were however expanded markedly in *RIP-mOVA:OTII:Rag2* ^−/−^ mice (Figure 3A). This was accompanied by a strong increase in total mTEC numbers (Figure 3B). CD4^+^ thymocytes in both *OTII:Rag2* ^−/−^ and *RIP-mOVA:OTII:Rag2* ^−/−^ mice upregulated expression of the chemokine receptor CCR7, enabling their migration into the medulla, where its ligand CCL21 is produced (data not shown). Positive selection also induced similar upregulation in TCR and CD3 expression in the two strains of mice (data not shown). Differential medulla expansion in *OTII:Rag2* ^−/−^ and *RIP-mOVA:OTII:Rag2* ^−/−^ mice could thus not be explained by differences in migration of CD4^+^ thymocytes into the medulla or in TCR upregulation upon positive selection.

Analysis of two other strains of TCR-transgenic mice, namely *BBM74:Rag2* ^−/−^ 28 and *B3K508:Rag1* ^−/−^ 29 mice, confirmed that positively-selected CD4^+^ thymocytes are not sufficient for inducing medulla expansion in the absence of cognate Ag. In both strains, medullas were as underdeveloped as in *OTII:Rag2* ^−/−^ mice (Figure S2A). Furthermore, leaky development of CD4^+^ thymocytes bearing non-transgenic TCRs in OTII:Rag2^+/+^ mice (∼1.7% of total CD4^+^ thymocytes, ∼3.5±0.4×10^5^ cells) was sufficient to drive WT medulla formation and mTEC development (Figure S2B–C). Low numbers of polyclonal CD4^+^ thymocytes comprising autoreactive specificities thus suffice to sustain medulla formation.

We previously established that the development of mature Aire^+^ mTECs could be driven by WT CD4^+^ thymocytes in reaggregated thymic organ culture (RTOC) experiments 14. We therefore used RTOC to determine whether Ag-specific interactions with CD4^+^ thymocytes were required for promoting mTEC development *in vitro*. 2-dGUO-treated fetal thymic stroma was reaggregated with *OTII:Rag2* ^−/−^ thymocytes in the presence or absence of OVA peptide (OVAp) (Figure 3C–D). mTEC development remained at baseline levels in RTOCs performed with OTII thymocytes in the absence of OVAp. In contrast, the addition of OVAp induced strong increases in the frequencies and numbers of total and mature mTECs, reaching levels equivalent to those induced by WT thymocytes.

**Figure 4:**
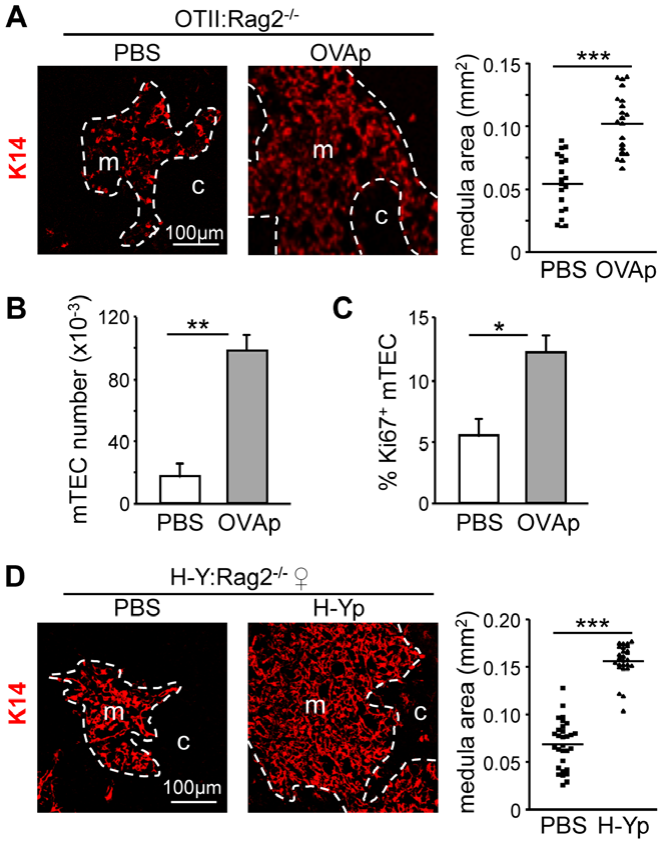
Ag-injection restores medulla formation in TCR transgenic mice. (A) Thymic sections from *OTII:Rag2* ^−/−^ mice injected with PBS or OVAp were stained with antibodies against K14: m, medulla; c, cortex. The graph shows medullary areas: data from 3 experiments, each with 3 mice per group; symbols represent individual confocal images; lines represent medians. (B) The graph shows numbers per thymus of CD45^−^EpCAM^+^Ly51^−/lo^ mTECs in *OTII:Rag2* ^−/−^ mice injected with PBS or OVAp. (C) The graph shows percentages of Ki67^+^ mTECs in *OTII:Rag2* ^−/−^ mice injected with PBS or OVAp: means and SD from 3 experiments, each with 3 mice per genotype. (D) Thymic sections from *Marilyn:Rag2* ^−/−^ females injected with PBS or H-Yp were stained with antibodies against K14: m, medulla; c, cortex. The graph shows medullary areas: data from 3 experiments, each with 3 mice per group; symbols represent individual confocal images; lines represent medians.

To determine whether medullary defects in TCR-transgenic mice can be corrected *in vivo* by providing the cognate Ag, we injected OVAp into adult *OTII:Rag2* ^−/−^ mice. After 5–7 days, medullary areas were strongly enlarged compared to PBS-injected controls (Figure 4A and S3A). This was accompanied by marked increases in total mTEC numbers (Figure 4B) and frequencies of proliferating Ki67^+^ mTECs (Figure 4C). OVAp injection also induced strong increases in mature mTECs, with enhanced Aire and Aire-dependent TRA expression (Figure S3B). 3D reconstructions of thymic lobes confirmed that medullary volume was increased in the OVAp-injected mice (1.4%) compared to PBS-injected controls (0.7%) (Figure S4A, movies S1, S2). In the OVAp-injected mice, individual medullary islets were reduced in number and had a larger size distribution (Figure S4B). OVAp injection thus led to marked remodeling of the 3D organization of the medulla. Similar results were obtained by injecting the H-Y Dby peptide (H-Yp) into *Marilyn*:*Rag2* ^−/−^ females (Figure 4D, Figure S4C–D, movies S3, S4).

Taken together, these results establish that autoreactive CD4^+^ thymocytes control development and organization of the thymic medulla in an Ag-dependent manner. This dynamic homeostatic process operates in adult mice and controls mTEC proliferation, medullary growth and 3D patterning of the medulla.

### Ltβr-Signaling Controls Mtec Cellularity In Synergy With Rank And Cd40

**Figure 5:**
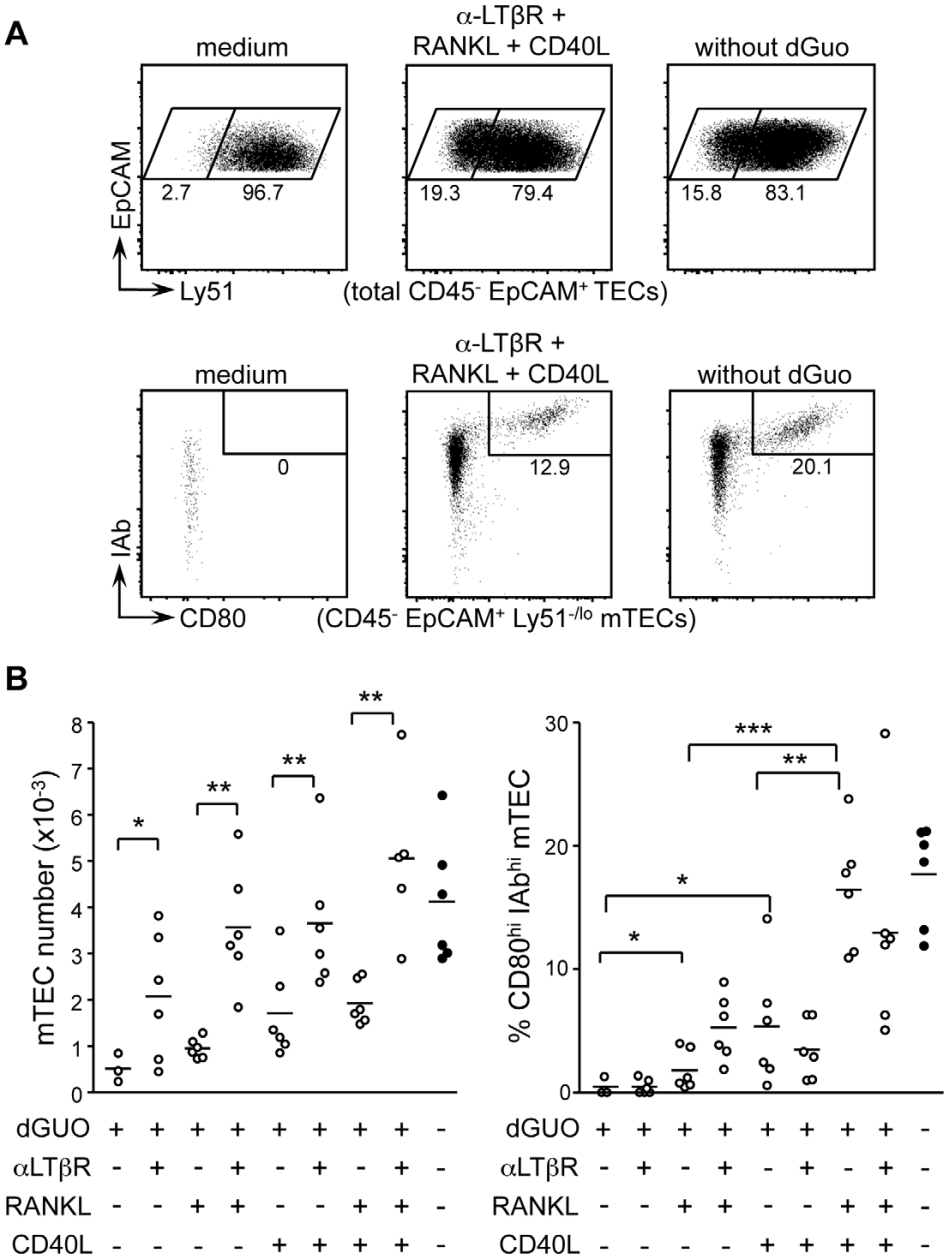
Roles of LTβR, RANK and CD40 signaling in mTEC expansion and maturation. 2-dGUO-treated WT embryonic thymic lobes were cultured for 4 days in medium containing agonistic anti-LTβR antibodies, CD40L and/or RANKL. Control cultures were un-supplemented (medium) or not treated with 2-dGUO. (A) Representative FACS profiles are shown for Ly51 expression by CD45^−^EpCAM^+^ TECs (top) and I-Ab and CD80 expression by CD45^−^EpCAM^+^Ly51^−/lo^ mTECs (bottom) for the indicated cultures: percentages of cells are indicated. (B) Graphs show mTEC numbers (left) and frequencies of mature I-Ab^hi^CD80^hi^ mTECs (right) for the indicated conditions: data from 3 experiments; lines represent medians.

Although it has been established that RANKL-RANK, CD40L-CD40 and LT-LTβR signaling contribute to mTEC development [14],[15],[16],[17],[19], the respective role of these three signaling axes remain unclear. To investigate this question, 2-dGUO-treated thymic lobes were cultured with agonistic anti-LTβR antibodies, CD40L and/or RANKL (Figure 5). Anti-LTβR antibodies induced a significant increase in total mTEC numbers. Little increase was induced by RANKL and CD40L, alone or in combination. Synergistic increases in mTEC numbers were observed when anti-LTβR antibodies were combined with RANKL, CD40L or both. Combining all 3 stimuli induced total mTEC numbers equivalent to those induced by thymocytes. A different division of labor emerged when mature mTECs were quantified (Figure 5A–B). RANKL and CD40L each induced a modest increase in the proportion of mature mTECs. A synergistic increase was obtained when RANKL and CD40L were added together, attaining control levels induced by thymocytes. In contrast, anti-LTβR antibodies had little or no influence on mature mTEC frequencies, either when added alone or in combination with RANKL, CD40L or both. Signaling via LTβR, CD40 and RANK thus make distinct contributions to mTEC cellularity and maturation. LTβR-signaling has a dominant role in determining total mTEC cellularity. Conversely, CD40 and RANK are critical for driving the development of mature mTEC.

### Lt Expression Is Regulated In Cd4^+^ Thymocytes By Ag-Specific Interactions With Mtecs

**Figure 6:**
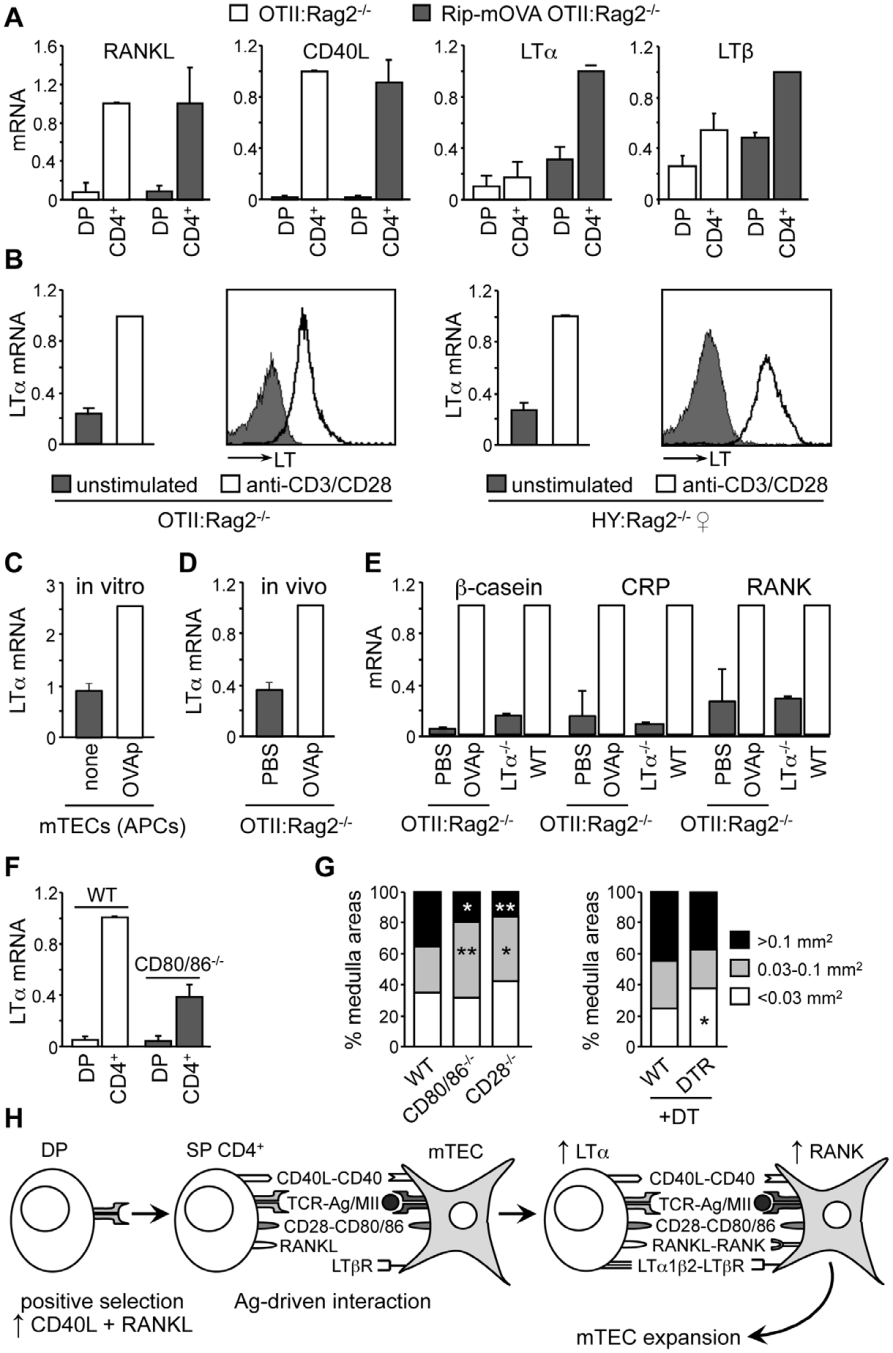
LT expression is induced by Ag-specific activation of CD4^+^ thymocytes. (A) RANKL, CD40L, LTα and LTβ mRNAs were quantified in DP and CD4^+^ thymocytes from *OTII:Rag2* ^−/−^ and *Rip-mOVA:OTII:Rag2* ^−/−^ mice: means and SEM are from 3 experiments, each with 2 mice per group. (B) LTα mRNA and cell surface LT were assessed for unstimulated and anti-CD3/CD28-activated CD4^+^ thymocytes from *OTII:Rag2* ^−/−^ or *Marilyn:Rag2* ^−/−^ mice: data representative of 3 experiments. (C) LTα mRNA was quantified in CD4^+^ thymocytes from *OTII:Rag2* ^−/−^ mice co-cultured with unloaded (none) or OVAp-loaded mTECs: data representative of 2 experiments. (D) LTα mRNA was quantified in CD4^+^ thymocytes from *OTII:Rag2* ^−/−^ mice isolated 1.5 days after injection of PBS or OVAp: data representative of 3 experiments. (E) β-casein, CRP and RANK mRNAs were quantified in mTECs from WT, LTα^−/−^ mice and *OTII:Rag2* ^−/−^ mice 5 days after injection of PBS or OVAp. (F) LTα mRNA was quantified in DP and CD4^+^ thymocytes from CD80/86^−/−^ mice: means and SEM are derived from 2 experiments, each with 2 mice per group. (G) Graphs show distributions of medullary areas (mm^2^) in WT, CD80/86^−/−^ and CD28^−/−^ thymi (left), and thymi from DT-treated WT and Foxp3-DTR mice (right): significance relative to WT. (H) Positive selection induces CD40L and RANKL expression in thymocytes. After migrating into the medulla, CD4^+^ thymocytes scan the surface of mTECs for the presence of auto-Ag–MHCII complexes. Ag-specific and CD28-CD80/86 dependent interactions between CD4^+^ thymocytes and mTECs induce the expression of LT in CD4^+^ thymocytes and RANK in mTECs, thereby completing the signaling axes required for promoting mTEC expansion and maturation.

To determine whether Ag-specific interactions with autoreactive CD4^+^ thymocytes might play a dominant role in inducing signals governing mTEC development, RANKL, CD40L, LTα and LTβ mRNAs were quantified by qRT-PCR in DP and CD4^+^ thymocytes from *OTII:Rag2* ^−/−^ and *Rip-mOVA:OTII:Rag2* ^−/−^ mice. In both strains, RANKL and CD40L mRNAs were upregulated strongly in CD4^+^ thymocytes relative to DP thymocytes. LTβ mRNA was also upregulated, albeit only ∼2-fold, in CD4^+^ thymocytes from both strains (Figure 6A). RANKL, CD40L and LTβ mRNAs were thus induced in positively-selected OTII thymocytes independently of OVA expression. In contrast, LTα mRNA expression was upregulated only in CD4^+^ thymocytes of *Rip-mOVA:OTII:Rag2* mice, suggesting that its induction occurs upon Ag-specific interactions with mTECs.

Three approaches confirmed that LT expression is induced in CD4^+^ thymocytes by TCR engagement. First, LTα mRNA and cell-surface LT expression were upregulated by activating CD4^+^ thymocytes from *OTII:Rag2* ^−/−^ mice or *Marilyn:Rag2* ^−/−^ females with anti-CD3/CD28 antibodies (Figure 6B). Second, LTα mRNA was upregulated selectively in OTII*:Rag2* ^−/−^ thymocytes co-cultured with OVAp-loaded mTECs (Figure 6C). Finally, injection of *OTII:Rag2* ^−/−^ mice with OVAp induced LTα mRNA expression in CD4^+^ thymocytes *in vivo* (Figure 6D). This Ag-induced LT expression had functional consequences at the level of mTECs, since LT-dependent TRA and RANK expression were strongly induced in mTECs of OVAp-injected mice (Figure 6E). TRA and RANK expression was impaired to the same extent in mTECs from PBS-injected *OTII:Rag2* ^−/−^ and LTα^−/−^ mice, suggesting that autoreactive CD4^+^ thymocytes constitute the major source of LT.

To confirm the role of physical interactions between CD4^+^ thymocytes and mTECs in controlling LT expression and medulla formation, we analyzed involvement of the CD28-CD80/86 costimulatory pathway. Upregulation of LTα mRNA was significantly impaired in CD4^+^ thymocytes of CD80/86^−/−^ mice (Figure 6F). The size distribution of medullary areas was skewed towards smaller sizes in CD80/86^−/−^ and CD28^−/−^ mice (Figure 6G), as previously described for LTα^−/−^ mice 20,21. Impaired medulla formation in CD80/86^−/−^ and CD28^−/−^ mice does not seem to result from abrogated natural regulatory T cell development 41,42, since no major reduction in medullary size was induced by regulatory T cell ablation in diphtheria-toxin treated Foxp3-DTR mice (Figure 6G). This suggests that conventional autoreactive CD4^+^ thymocytes, rather than natural regulatory T cells, are the major actors in regulating medulla formation.

## Discussion

We investigated the respective contributions of CD4^+^ and CD8^+^ thymocytes to formation and patterning of the thymic medulla. Mice lacking CD8^+^ thymocytes exhibited normally sized medullary islets and WT mTEC numbers. In contrast, mice lacking CD4^+^ thymocytes exhibited poorly developed medullary islets and strongly reduced mTEC cellularity. CD4^+^ thymocytes are thus essential and sufficient for driving proper medulla growth, whereas CD8^+^ thymocytes are dispensable for sustaining this process. Despite this privileged role of CD4^+^ thymocytes, medulla formation was severely impaired in MHCII-restricted TCR-transgenic mice in which positive selection of CD4^+^ thymocytes occurs normally while there is no negative selection since the cognate Ag is absent. RTOC experiments also demonstrated that OTII thymocytes were unable to drive mTEC development in the absence of OVAp. Positively-selected CD4^+^ thymocytes are thus not sufficient *per se* for sustaining medulla formation. Instead, several lines of evidence indicate that medullary growth requires Ag-specific interactions between autoreactive CD4^+^ thymocytes and mTECs, similar to those that induce negative selection. First, OTII thymocytes can promote mTEC development in RTOCs as efficiently as WT thymocytes when OVAp is provided. Second, whereas mTEC cellularity was strongly decreased in *OTII:Rag2* ^−/−^ mice and *Marilyn*:*Rag2* ^−/−^ females, this defect was corrected in *RIP-mOVA:OTII:Rag2* ^−/−^ mice and *Marilyn*:*Rag2* ^−/−^ males, which express the cognate Ags. Third, medullary defects in *OTII:Rag*2^−/−^ mice and *Marilyn*:*Rag*2^−/−^ females could be corrected by injecting OVAp and H-Yp, respectively. The latter finding shows that defective medulla formation in these mice is not simply due to an intrinsic developmental block. It also emphasizes the remarkable plasticity of the medulla and indicates that the control of its size by autoreactive CD4^+^ thymocytes is a dynamic homeostatic process operating in adult mice. This is consistent with studies indicating that medullary defects in adult SCID mice can be corrected by injecting mature T cells 43,44. Fourth, rare CD4^+^ thymocytes with non-transgenic TCR specificities in *OTII:Rag* ^+/+^ mice suffice to sustain medulla formation, suggesting that this process is sensitive to low numbers of autoreactive CD4^+^ thymocytes. This is consistent with the fact that SP thymocytes reside in the medulla for 4–5 days and are highly mobile, favoring numerous encounters with mTECs 45,46.

We exploited *OTII:Rag2* ^−/−^ and *RIP-mOVA:OTII:Rag2* ^−/−^ mice to determine whether upregulation of LT, RANKL and CD40L expression in SP CD4^+^ thymocytes might be driven by Ag-specific interactions with mTECs. LTβ, RANKL and CD40L mRNAs were upregulated independently of OVA expression in both *OTII:Rag2* ^−/−^ and *RIP-mOVA:OTII:Rag2* ^−/−^ CD4^+^ thymocytes. Conversely, increased LTα mRNA expression was dependent on OVA expression by mTECs, as it was only observed in RIP-*mOVA:OTII:Rag2* ^−/−^ mice. Upregulation of LT expression by TCR stimulation was confirmed *in vitro* by activating OTII thymocytes with anti-CD3/CD28 antibodies or by co-culture with OVAp-loaded mTECs. These results suggest that expression of RANKL and CD40L is induced by positive selection in the cortex whereas that of LT is activated by subsequent Ag-specific interactions with mTECs (Figure 6H). Activation of LT expression is thus uncoupled physically and temporally from the induction of RANKL and CD40L expression.

Cooperation between RANKL and CD40L signaling has been shown to be critical for mTEC development in the postnatal thymus 16,17. However, RANKL and CD40L expression by CD4^+^ thymocytes is not sufficient because mTEC development is severely impaired in OTII:Rag2^−/−^ mice even though their thymocytes express both ligands. This could be reconciled by three non-mutually-exclusive mechanisms. First, efficient delivery of RANKL and CD40L signals to mTECs may require stable Ag-driven contacts with CD4^+^ thymocytes. Second, effective control of mTEC development by RANKL and CD40L requires collaboration with LT expression, which is induced in CD4^+^ thymocytes by Ag-dependent interactions with mTECs. Finally, LT produced by autoreactive CD4^+^ thymocytes enhances the responsiveness of mTECs to RANKL signals by inducing RANK expression in mTECs (Figure 6H).

Our FTOC experiments indicated that LT, RANKL and CD40L signals make differential contributions to mTEC expansion and maturation. LTβR signaling was critical for fostering an increase in mTEC cellularity but had little effect on mTEC maturation. A key role of LTβR signaling in mTEC expansion is consistent with the observation that LTβR^−/−^ and LTα^−/−^ mice have small medullas 19,20,21 whereas LT over-expression in T cells leads to drastic medulla enlargement 47. In contrast to LTβR signaling, synergy between RANKL and CD40L was essential for driving mTEC maturation rather than increasing mTEC numbers. This is consistent with studies showing that mTEC maturation requires cooperation between RANKL and CD40L and that mTEC cellularity is decreased only modestly in RANKL^−/−^ and CD40^−/−^ mice 16,17.

In conclusion, at least four parameters - medullary size, mTEC numbers, 3D organization of the medulla and mTEC maturation - are modulated in adult mice by Ag-specific and costimulatory-molecule-dependent interactions between autoreactive CD4^+^ thymocytes and mTECs displaying auto-Ag-MHCII complexes (Fig. 6H). These interactions induce LT expression by autoreactive CD4^+^ thymocytes and RANK expression by mTECs, thereby completing key signaling axes required for medulla formation (Fig. 6H). This unique crosstalk between autoreactive CD4^+^ thymocytes and mTECs regulates a homeostatic fine-tuning process that controls mTEC cellularity and 3D organization of the postnatal medulla, thereby maintaining a medullary microenvironment that is optimal for ensuring central T-cell tolerance.

## Supporting Information

Figure S1 **mTECs but not DCs are impaired in mice lacking CD4^+^ thymocytes.** (A) Representative FACS profiles for the expression of Aire and CD80 by CD45^−^EpCAM^+^Ly51^−/lo^ mTECs from WT, *β2m* ^−/−^, *H2-Aα* ^−/−^ and *CIIta* ^IV-/IV-^ mice: numbers represent the percentages of cells within the indicated gates. Graphs show number of Aire^+^, CD80^hi^, CD80^int^ and CD80^lo^ mTECs: the means and SD were derived from three measurements, each with three mice per genotype; statistical significance relative to WT. (B) Representative FACS profiles for the expression of CD11c and PDCA1 by CD45^+^ hematopoietic cells (top profiles), and the expression of Sirpα and CD8α by CD45^+^CD11c^hi^ cDCs (bottom profiles), are shown for thymi from WT, *β2m* ^−/−^, *H2-Aα* ^−/−^ and *CIIta* ^IV-/IV-^ mice. Graphs show numbers per thymus of CD11c^int^PDCA1^+^ pDCs, CD11c^hi^CD8α^lo^Sirpα^+^ cDCs and CD11c^hi^CD8α^hi^Sirpα^−^ cDCs for the indicated genotypes: the means and SEM are derived from two experiments, each with four to seven mice per group. Numbers of mice is indicated. (C) Thymic sections from WT, *β2m* ^−/−^, *H2-Aα* ^−/−^ and *CIIta* ^IV-/IV-^ mice were stained for the CD11c marker: m denotes the medulla. Results are representative of two experiments, each with two mice per group. (TIFF)

Figure S2 **Positively-selected CD4^+^ thymocytes in** ***3BBM74:Rag2*** **^−/−^,** ***B3K508:Rag1*** **^−/−^ and** ***OTII:Rag2*** **^−/−^ are inefficient at inducing medulla expansion whereas low numbers of autoreactive CD4^+^ thymocytes are sufficient.** (A) Thymic sections from *3BBM74:Rag2* ^−/−^, *B3K508:Rag1* ^−/−^, *OTII:Rag2* ^−/−^ and WT mice were stained with antibodies against K8 and K14: m denotes the medulla; c denotes the cortex. Graphs show quantifications of the K14^+^ areas (left graph) and medullary areas (right graph) for each genotype: symbols represent individual confocal images; horizontal lines represent medians; data is pooled from three independent experiments, each with two to three mice per group. (B) Graphs show quantifications of the medullary areas, K14^+^ and MTS10^+^ areas in thymic sections from *OTII:Rag2* ^−/−^, *OTII:Rag2* ^+/+^ and WT mice: symbols represent individual confocal images; horizontal lines represent medians; data was pooled from three independent experiments, each with three mice per group. (C) Representative FACS profiles are shown for the expression of Ly51 by CD45^−^EpCAM^+^Ly51^−/lo^ mTECs from *OTII:Rag2* ^−/−^, *OTII:Rag2* ^+/+^ and WT mice: numbers indicate percentages of cells within the indicated gates. Graphs show the percentages of mTECs and cTECs in the TEC population: the means and SD derived of three experiments are shown; statistical significance relative to WT. (TIFF)

Figure S3 **Restoration of thymic medulla formation in** ***Rag2*** **^−/−^ TCR transgenic mice by** ***i.v*** **injection of the cognate Ag.** (A) Thymic sections from *OTII:Rag2* ^−/−^ mice injected with PBS or OVA_323–339_ were stained with antibodies against MTS10: m and c denote the medulla and cortex. The graph shows quantifications of MTS10^+^ medullary areas: data is pooled from three independent experiments, each with three mice per group; symbols represent individual confocal images; horizontal lines represent the medians. (B) Representative FACS profiles for the expression of Aire and CD80 by CD45^−^EpCAM^+^Ly51^−/lo^ mTECs from *OTII:Rag2* ^−/−^ mice injected with PBS or OVA_323–339_: numbers indicate the percentage of cells within the indicated gates. Graphs show the frequencies and numbers of Aire^+^ and CD80^hi^ mTECs (means and SD derived from three experiments): number of mice is indicated. Aire and insulin mRNA expression were measured in mTECs pooled from 7 mice per group. (C) Thymic sections from *Marilyn:Rag2* ^−/−^ mice injected i.v with PBS or H-Y Dby peptide were stained with antibodies against MTS10: m and c denote the medulla and cortex. The graph shows MTS10^+^ medullary areas: data is pooled from three independent experiments, each with three mice per group; symbols represent individual confocal images; horizontal lines represent the medians. (TIFF)

Figure S4 **Injection of the cognate peptide induces expansion of the thymic medulla in** ***OTII:Rag2*** **^−/−^ mice and female** ***Marilyn*** **:** ***Rag2*** **^−/−^ mice.** (A) *OTII:Rag2* ^−/−^ mice were injected i.v with PBS or OVA_323–339_. 5 days later, 3D reconstructions of thymic lobes were generated from serial sections stained with antibodies against K14 and DAPI. 3D reconstructions depicting the thymic lobes (light blue, DAPI) and medulla (red, K14) are shown: axes are in mm. Volumes and percentages of the thymic medulla are indicated. (B) The graph depicts the volumes (mm^3^) of individual medullary islets in thymic lobes from *OTII:Rag2* ^−/−^ mice injected with PBS or OVA_323–339_. Horizontal lines represent medians and SEM. Numbers of isolated medullary islets in the thymic lobes are indicated below. The 3D reconstructions can be visualized in movies S1, S2. (C) Female *Marilyn*:Rag2^−/−^ mice were injected i.v with PBS or H-Y Dby peptide. 5 days later, 3D reconstructions of thymic lobes were then generated from serial sections stained with antibodies against K14 and DAPI. 3D reconstructions depicting the thymic lobes (light blue, DAPI) and medulla (red, K14) are shown: axes are in mm. Volumes and percentages of the thymic medulla are indicated. (D) The graph depicts the volumes (mm^3^) of individual medullary islets in thymic lobes from *Marilyn*:*Rag2* ^−/−^ mice injected with PBS or Dby H-Y peptide. Horizontal lines represent medians and SEM. Numbers of isolated medullary islets in the thymic lobes are indicated below. The 3D reconstructions can be visualized in movies S3, S4. (TIFF)

Movie S1 **3D reconstruction of an entire thymic lobe from** ***OTII:Rag2^−/−^*** **mouse injected with PBS.** Thymic and medullary volumes were reconstructed using a whole thymic lobe. 20 µm thick serial sections were stained with DAPI (light blue) and antibodies against K14 (red). Volume rendering was performed from epifluorescence images, as described in methods. All axes are graduated in mm. (AVI)

Movie S2 **3D reconstruction of an entire thymic lobe from** ***OTII:Rag2^−/−^*** **mouse injected with the OVA_323–339_ peptide.** Volumes were determined as described for Movie S1. (AVI)

Movie S3 **3D reconstruction of an entire thymic lobe from female** ***Marilyn*** **:** ***Rag2*** **^−/−^ mouse injected with PBS.** Volumes were determined as described for Movie S1. (AVI)

Movie S4 **3D reconstruction of an entire thymic lobe from female** ***Marilyn*** **:** ***Rag2*** **^−/−^ mouse injected with the H-Y Dby peptide.** Volumes were determined as described for Movie S1. (AVI)
