# Supplementary figures and images for: Antigen Recognition By Autoreactive Cd4+ Thymocytes Drives Homeostasis Of The Thymic Medulla
Source: PLoS One. 2012 Dec 27;7(12):e52591. doi: 10.1371/journal.pone.0052591 (PMC3531460; doi:10.1371/journal.pone.0052591)

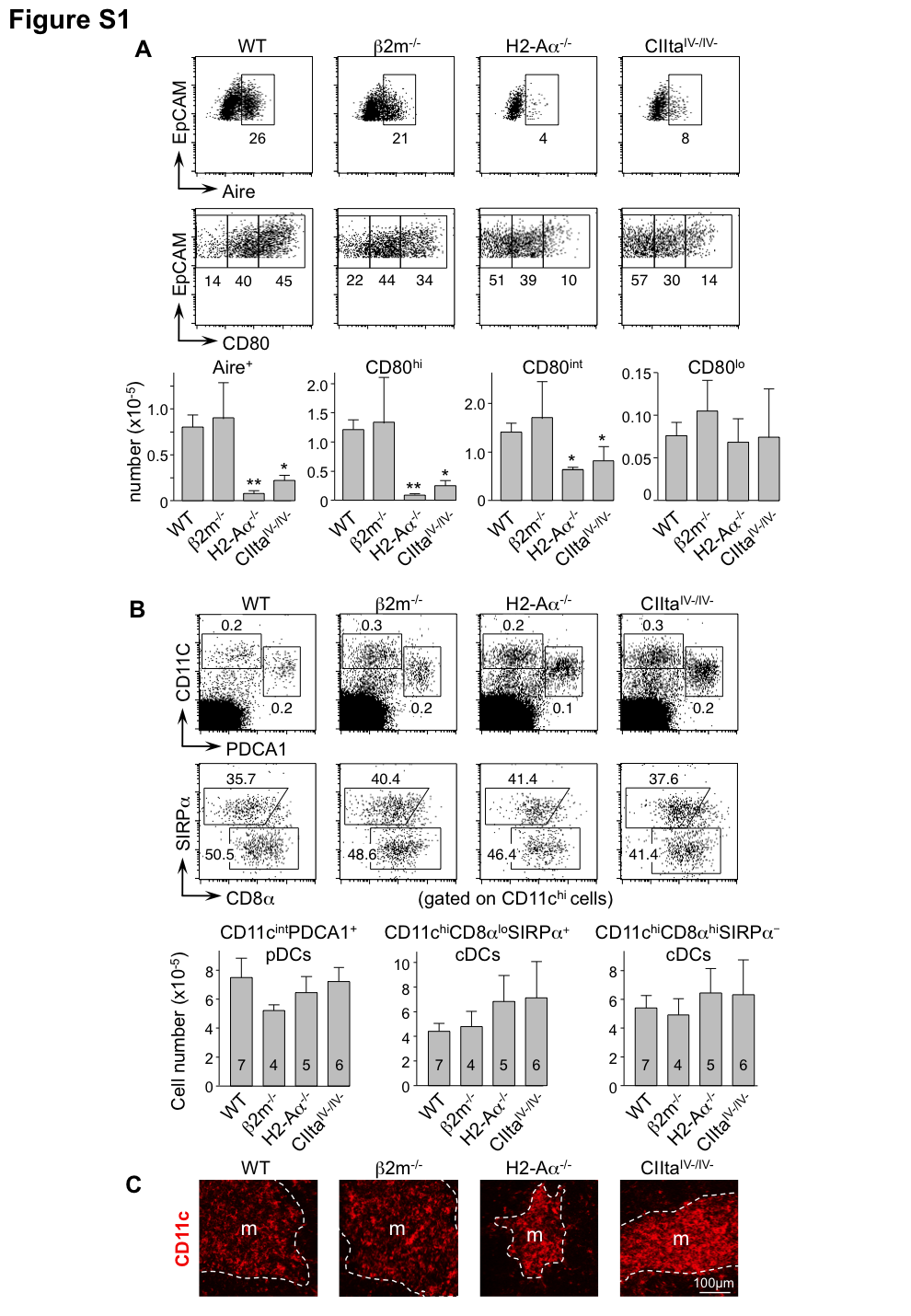

Supplement: Figure S1 — (TIFF) [file pone.0052591.s001.tiff]

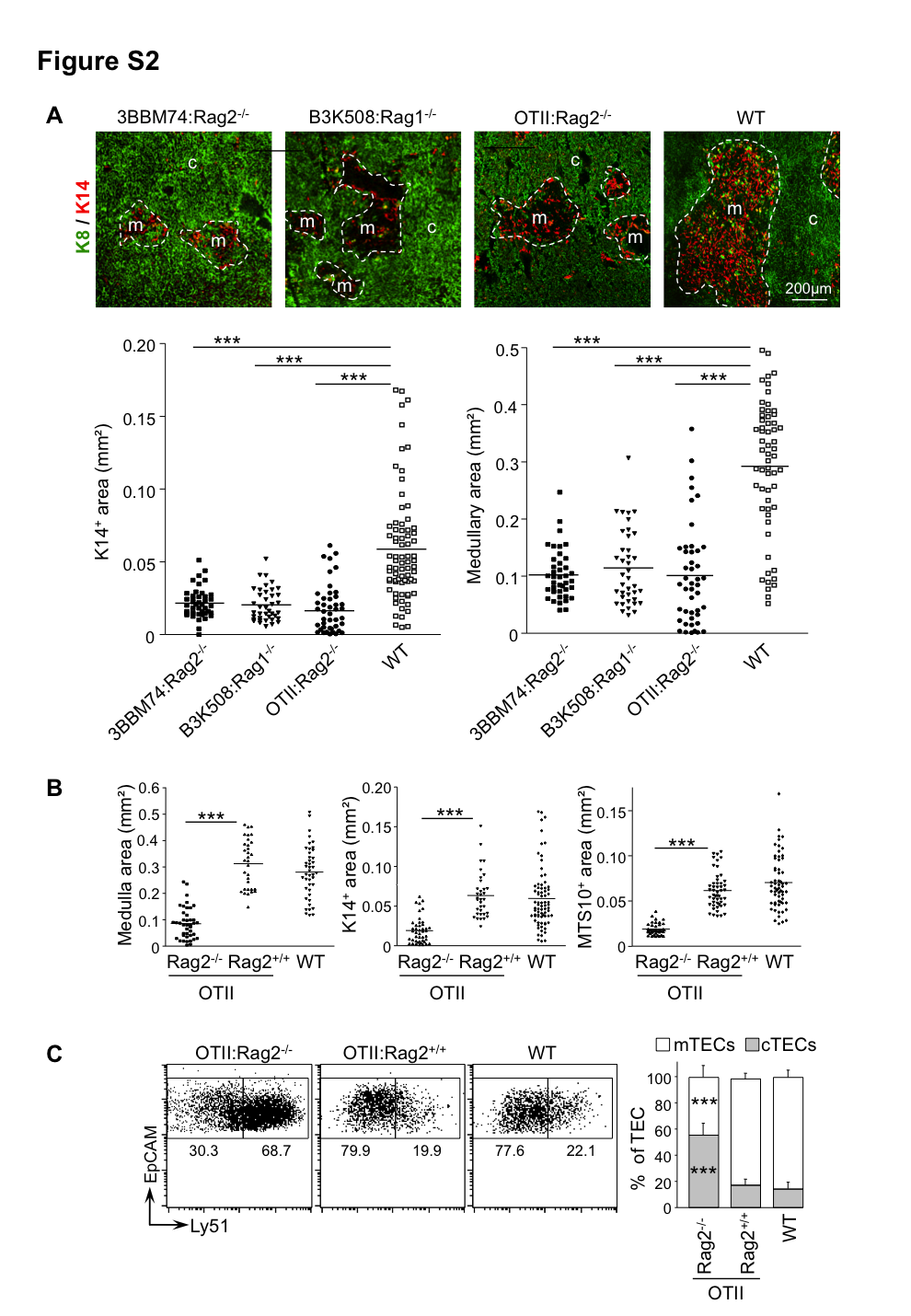

Supplement: Figure S2 — (TIFF) [file pone.0052591.s002.tiff]

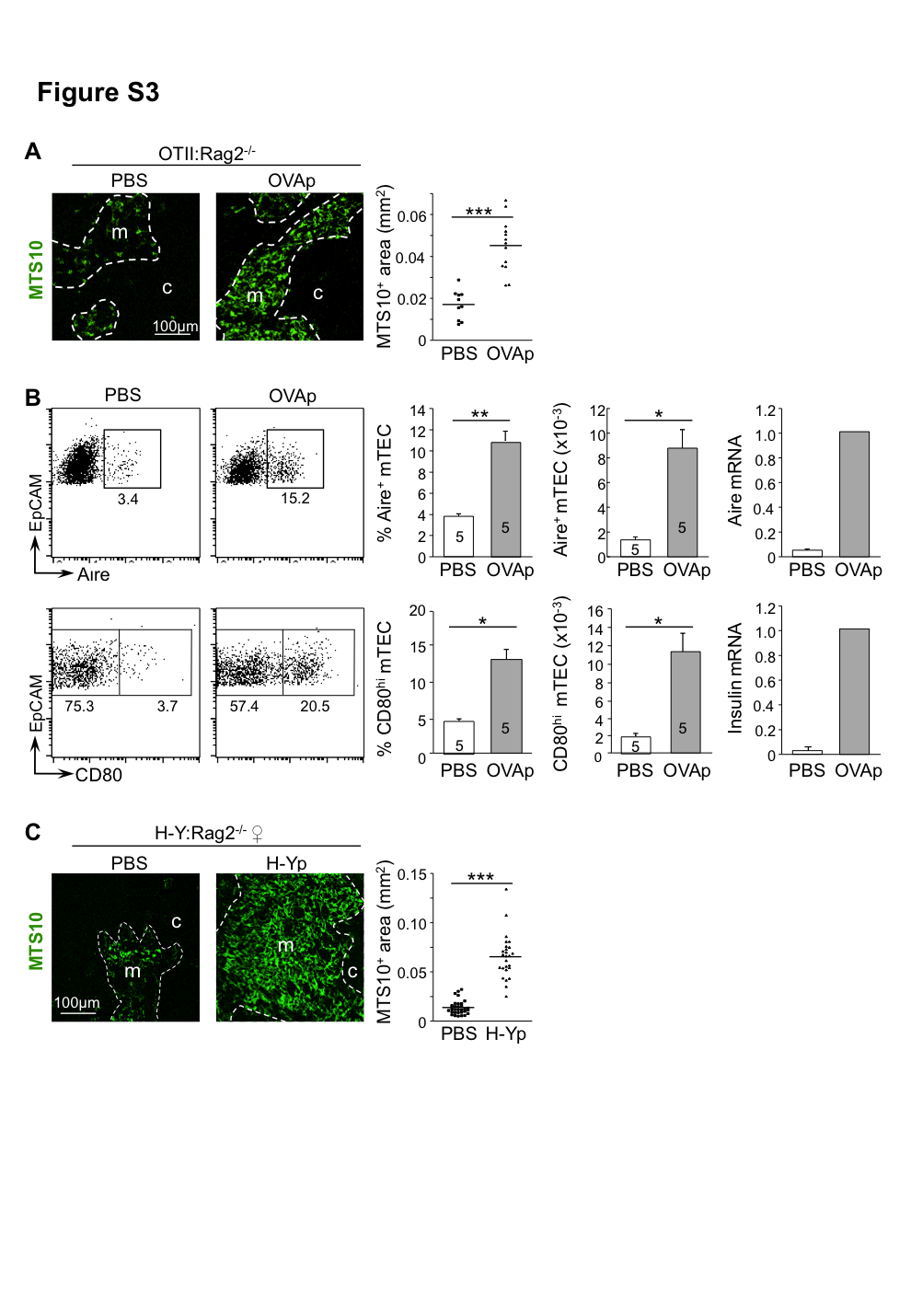

Supplement: Figure S3 — (TIFF) [file pone.0052591.s003.tiff]

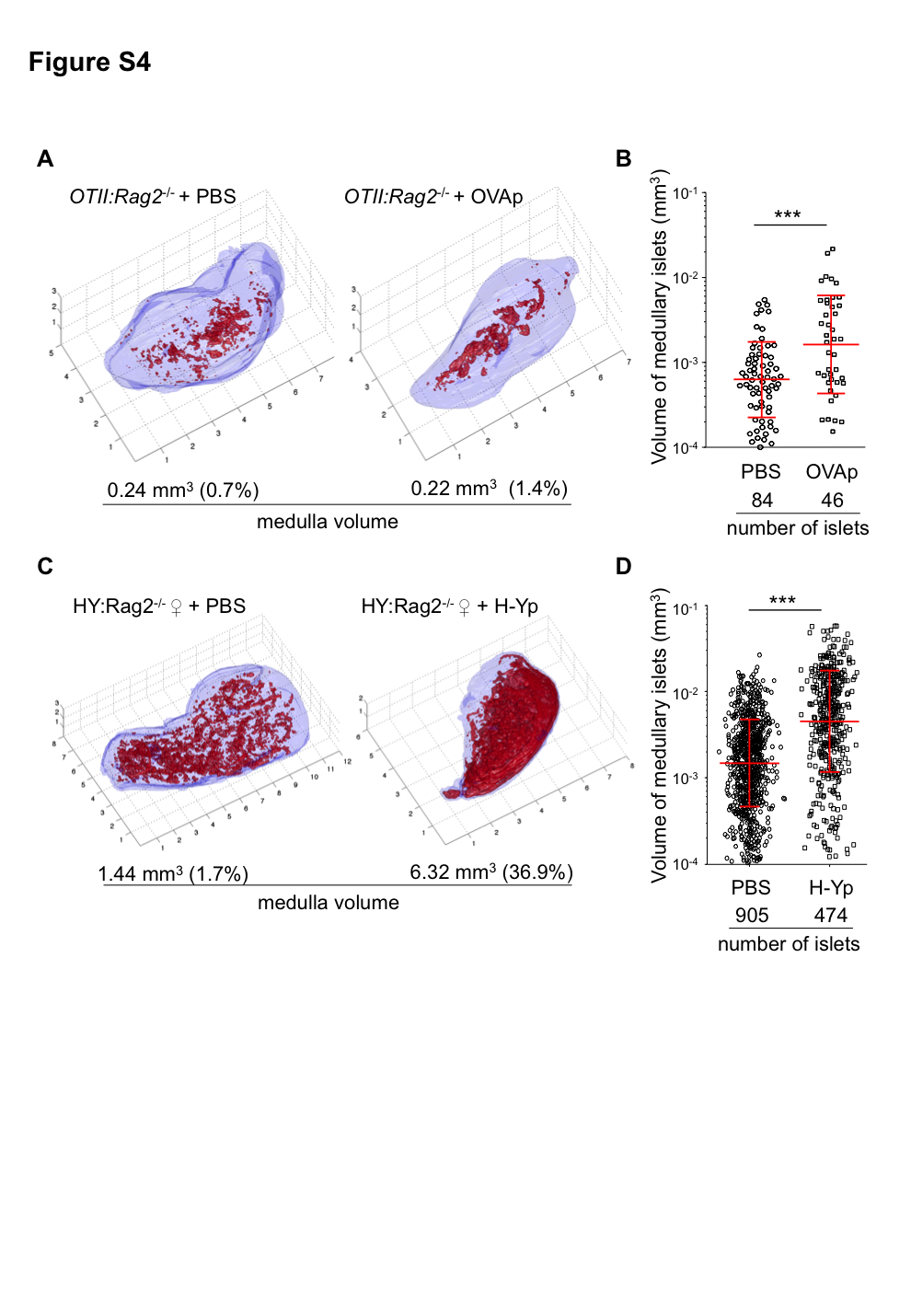

Supplement: Figure S4 — (TIFF) [file pone.0052591.s004.tiff]
